# Helios, and not FoxP3, is the marker of activated Tregs expressing GARP/LAP

**DOI:** 10.18632/oncotarget.4771

**Published:** 2015-07-30

**Authors:** Eyad Elkord, May Abd Al Samid, Belal Chaudhary

**Affiliations:** ^1^ College of Medicine & Health Sciences, United Arab Emirates University, Al Ain, United Arab Emirates; ^2^ Biomedical Research Centre, School of Environment & Life Sciences, University of Salford, Manchester, United Kingdom; ^3^ Institutes of Cancer, Inflammation & Repair, University of Manchester, Manchester, United Kingdom; ^4^ University of Cambridge, Cambridge, United Kingdom

**Keywords:** Immunology and Microbiology Section, Immune response, Immunity, regulatory T cells, GARP/LAP, FoxP3, Helios

## Abstract

Regulatory T cells (Tregs) are key players of immune regulation/dysregulation both in physiological and pathophysiological settings. Despite significant advances in understanding Treg function, there is still a pressing need to define reliable and specific markers that can distinguish different Treg subpopulations. Herein we show for the first time that markers of activated Tregs [latency associated peptide (LAP) and glycoprotein A repetitions predominant (GARP, or LRRC32)] are expressed on CD4^+^FoxP3^−^ T cells expressing Helios (FoxP3^−^Helios^+^) in the steady state. Following TCR activation, GARP/LAP are up-regulated on CD4^+^Helios^+^ T cells regardless of FoxP3 expression (FoxP3^+/−^Helios^+^). We show that CD4^+^GARP^+/−^LAP^+^ Tregs make IL-10 immunosuppressive cytokine but not IFN-γ effector cytokine. Further characterization of FoxP3/Helios subpopulations showed that FoxP3^+^Helios^+^ Tregs proliferate *in vitro* significantly less than FoxP3^+^Helios^−^ Tregs upon TCR stimulation. Unlike FoxP3^+^Helios^−^ Tregs, FoxP3^+^Helios^+^ Tregs secrete IL-10 but not IFN-γ or IL-2, confirming they are bona fide Tregs with immunosuppressive characteristics. Taken together, Helios, and not FoxP3, is the marker of activated Tregs expressing GARP/LAP, and FoxP3^+^Helios^+^ Tregs have more suppressive characteristics, compared with FoxP3^+^Helios^−^ Tregs. Our work implies that therapeutic modalities for treating autoimmune and inflammatory diseases, allergies and graft rejection should be designed to induce and/or expand FoxP3^+^Helios^+^ Tregs, while therapies against cancers or infectious diseases should avoid such expansion/induction.

## INTRODUCTION

Regulatory T cells (Tregs) are immunosuppressive cells that regulate function of the immune system. They are key in maintaining peripheral immune tolerance. Tregs have defined roles in various pathological settings including cancer, autoimmune diseases and transplant rejection, where they contribute to disease progression [1, 2]. Given their role in immune modulation under pathological conditions, Tregs have increasingly been targeted for novel immunotherapies with some promising results in clinical trials [3, 4]. Current approaches include Treg depletion and functional blockade to enhance immune responses or autologous transfer of Tregs to dampen immune responses in certain scenarios [3–5].

Tregs are broadly divided into thymic-derived Tregs (tTregs) and peripheral-induced Tregs (pTregs). Both of these Treg subsets were originally defined as expressing the forkhead box P3 transcription factor (FoxP3) and the alpha chain of the IL-2 receptor (CD25). FoxP3 is crucial for development and maintenance of the suppressive Treg lineage [2]. pTregs comprise two further FoxP3^−^ subsets; IL-10 secreting Type 1 Tregs (Tr1) and T helper 3 Tregs (Th3) [6, 7]. Recent years have seen significant efforts into characterizing Treg markers for effective identification and isolation of Treg subsets [2, 5]. The expression of FoxP3, CD25 or the ‘immune checkpoint’ molecules proposed to identify Tregs, can also define non-regulatory T cells (Tconv) and effector T cells (Teff) [5, 8]. CD25 is a well-recognized T cell activation marker. The immune checkpoint molecules, CTLA-4, PD-1, LAG-3 and TIM-3, can also be up-regulated on activated Tconv and Teff [5]. Neuropilin 1 (NRP1), a promising Treg surface marker, is also up-regulated during inflammation *in vivo* [9, 10]. FoxP3 can be transiently expressed in T cells undergoing activation or in inflammatory microenvironments [11, 12].

We have recently reviewed current Treg markers [5]. Of particular interest are two molecules involved in the expression and activation of transforming growth factor-beta (TGF-β) that selectively identify activated Tregs: glycoprotein A repetitions predominant (GARP/LRRC32) and latency-associated peptide (LAP). LAP and TGF-β form inactive complexes on the surfaces of T cells, known as latent TGF-β complexes. These complexes can be cleaved to release active TGF-β. GARP plays a critical role in the formation and expression of latent TGF-β complexes at the cell surface by anchoring the complexes to the cell membrane [13, 14].

GARP is a transmembrane protein that has been identified on activated Tregs, megakaryocytes and platelets [13, 15, 16]. GARP forms a positive-feedback loop with FoxP3 where expression of one enhances expression of the other [16, 17]. Retroviral expression of GARP on human T helper (Th) cells with TCR stimulation results in stable re-programming of Th cells into functionally suppressive FoxP3-expressing Tregs [16, 18]. Down-regulation or silencing of GARP on FoxP3^+^ Tregs decreases both FoxP3 expression and suppressive activity [16, 18]. Induction of GARP on naïve human T cells also induced partial Treg functionality *in vitro* [19]. GARP itself might contribute directly to immune suppression as shown in a humanized mouse model where treatment with soluble GARP (sGARP) prevented lethal graft-versus-host disease (GVHD) following xenogeneic tissue transplantation [20]. sGARP also induced development of naïve CD4^+^ human T cells into Tregs *in vitro* [20].

LAP is up-regulated on activated Tregs and has been characterized on megakaryocytes and immature dendritic cells (DCs) [21]. LAP has been utilized in conjunction with the IL-1 receptors Type I/II (CD121a/b) for identification and isolation of functional FoxP3^+^ human Tregs [22]. Highly suppressive FoxP3^−^LAP^+^ Tregs have also been identified in humans [23]. LAP was recently used to effectively identify and isolate functional Tregs from patients following immunotherapy with anti-CTLA-4 antibody [24].

Another marker that has been the focus of significant research is the Ikaros zinc finger transcription factor, Helios. It was first identified as a selective tTreg marker in mice [25]. However, in humans, Helios has been characterized in pTregs, tTregs, CD8^+^ T cells, activated T cells and T cells in inflammatory microenvironments [5, 26]. Helios has been implicated in Treg development and stability by repressing the IL-2 gene promoter [27, 28]. Helios^+^ Tregs have been shown to exhibit superior suppressive activity, compared to Helios^−^ Tregs in mice [29]. In human Tregs, Helios has been reported to enhance FoxP3 expression by binding the FoxP3 promoter [30]. Helios knockdown impaired the suppressive activity and down regulated FoxP3 expression [30]. While the exact role of Helios is uncertain, it is acceptable that Helios defines a highly suppressive Treg subset with distinct phenotypic and functional features [31–33]. FoxP3^+^Helios^+^ Tregs are significantly expanded in circulation and tumor microenvironment in various cancers including colorectal cancer, renal cell carcinoma and glioblastoma [34–37].

The correlation of FoxP3 and Helios with GARP and LAP has not been studied previously. Only one study reported that GARP did not correlate with Helios expression in CD4^+^ or in CD4^+^FoxP3^+^ cells [26]. LAP expression was not investigated. In this study, we investigated the co-expression of GARP and LAP with FoxP3 and Helios on T cells isolated from the peripheral blood of healthy donors. Herein, we show for the first time that markers of activated Tregs, GARP/LAP, are mainly expressed on FoxP3^+^Helios^+^ Tregs, but not on FoxP3^+^Helios^−^ Tregs. Additionally GARP and LAP are expressed on a FoxP3^−^Helios^+^ subset, indicating that Helios, but not FoxP3, is the marker of activated Tregs expressing GARP/LAP. We also report that FoxP3^+^Helios^+^ Tregs exhibit more immunosuppressive characteristics compared to FoxP3^+^Helios^−^ Tregs, further supporting the role of Helios as a marker of suppressive Tregs.

## RESULTS

### GARP/LAP are not expressed on FoxP3^+^ Tregs in steady state

**Figure 1:**
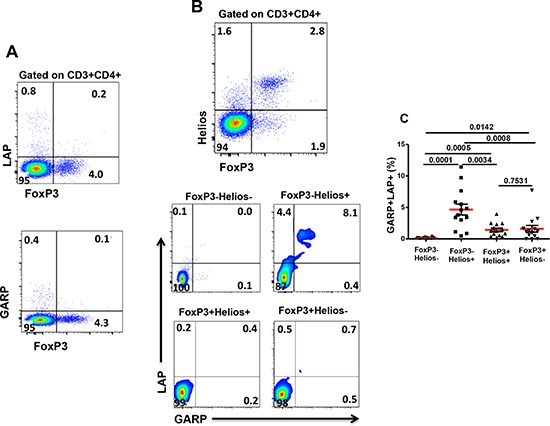
Expression of GARP and LAP on different FoxP3^+/−^Helios^+/−^ non-activated T-cell subsets Thawed PBMCs isolated from healthy donors were stained for CD3, CD4, GARP and LAP surface markers followed by FoxP3 and Helios intracellular staining. **A.** Representative flow cytometric plots showing FoxP3 expression against LAP or GARP, as gated on CD3^+^CD4^+^ T cells. **B.** Representative flow cytometric plots showing FoxP3^−^Helios^−^, FoxP3^−^Helios^+^, FoxP3^+^Helios^+^ and FoxP3^+^Helios^−^ T-cell subsets and the expression of GARP/LAP within these subsets in non-activated cells. **C.** Scatter plots show the mean percentage ± SEM of GARP^+^LAP^+^ within FoxP3^−^Helios^−^, FoxP3^−^Helios^+^, FoxP3^+^Helios^+^ and FoxP3^+^Helios^−^ T-cell subsets in non-activated PBMCs isolated from 14 healthy donors.

We investigated co-expression of different key Treg markers including FoxP3 and Helios, as master Treg transcription factors, and LAP and GARP, as markers of activated Tregs. In steady-state non-activated T cells from healthy donors, we noticed that LAP and GARP were not co-expressed with FoxP3 on CD4^+^ Tregs, and low levels (<1%) of LAP^+^ or GARP^+^ cells were detected in CD4^+^ T cells lacking FoxP3 expression (Figure 1A). This is consistent with a recent observation in patients with acute coronary syndrome [38].

### GARP/LAP are expressed on a subset of CD4^+^FoxP3^−^Helios^+^ in non-activated setting

To further define Treg subpopulations and investigate GARP/LAP co-expression, we combined FoxP3 and Helios staining and analyzed the co-expression of GARP/LAP on these different subsets. When CD4^+^Helios^+^ T cells were gated, we found that some cells within this subset express GARP/LAP. Of note, a significant percentage of CD3^+^CD4^−^ (CD8^+^) T cells expresses Helios but unlike CD4^+^Helios^+^ T cells, there is no GARP/LAP expressed on CD8^+^ T cells (not shown).

Different FoxP3^+/−^Helios^+/−^ CD4^+^ T cell subsets were gated, as shown in Figure 1B (first plot). GARP/LAP were expressed at negligible levels on non-activated CD4^+^FoxP3^+^ Tregs, regardless of Helios expression (Figure 1B, last two plots). Interestingly, the only subpopulation that expressed significantly higher levels of GARP/LAP (4.7 ± 0.8%), compared with other subpopulations, was CD4^+^FoxP3^−^Helios^+^ in non-activated setting (Figure 1B & 1C).

### GARP/LAP are expressed on Helios^+^, regardless of FoxP3 expression, in activated CD4^+^ T cells

**Figure 2:**
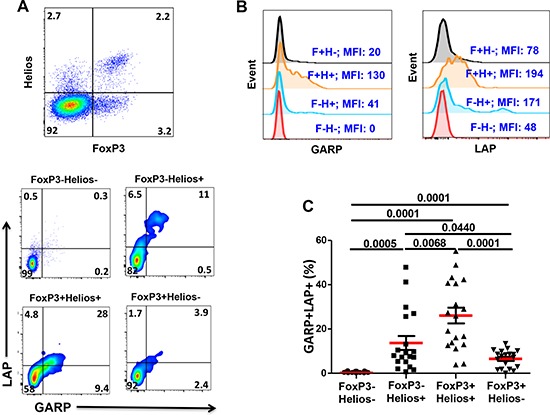
Expression of GARP and LAP on different FoxP3^+/−^Helios^+/−^ T-cell subsets in the activated setting PBMCs from healthy donors were activated by plate-bound anti-CD3/28 followed by surface staining for CD3, CD4, GARP and LAP and intracellular staining for FoxP3 and Helios. **A.** Representative flow cytometric plots showing FoxP3^−^Helios^−^, FoxP3^−^Helios^+^, FoxP3^+^Helios^+^ and FoxP3^+^Helios^−^ T-cell subsets and the expression of GARP/LAP within these subsets in activated PBMCs. **B.** Representative overlaid histogram plots show the MFIs of GARP and LAP within the different FoxP3/Helios subsets. **C.** Scatter plots show the mean percentage ± SEM of GARP^+^LAP^+^ within FoxP3^−^Helios^−^, FoxP3^−^Helios^+^, FoxP3^+^Helios^+^ and FoxP3^+^Helios^−^ T-cell subsets in activated PBMCs isolated from 19 healthy donors.

We then investigated GARP/LAP expression on FoxP3/Helios CD4^+^ T cell subsets following TCR stimulation for 18–20 hours. Different FoxP3^+/−^Helios^+/−^ CD4^+^ T cell subsets were gated, as shown in Figure 2A (first plot). As expected, GARP/LAP were expressed at much higher levels on activated CD4^+^ T cells, compared with non-activated CD4^+^ T cells (Figures 1B & 2A). Figure 2A & 2B show the percentages of CD4^+^ T cells expressing GARP/LAP and the Mean Fluorescence Intensities (MFIs) of GARP and LAP expression within the different subsets. Interestingly, GARP/LAP were mainly expressed on FoxP3^+^Helios^+^ (26.1 ± 3.8%) and FoxP3^−^Helios^+^ (13.7 ± 3.2%) and to a significantly lower level on FoxP3^+^Helios^−^ (6.5 ± 0.9%), but not on FoxP3^−^Helios^−^ (0.4 ± 0.1%) CD4^+^ T cells (Figure 2C).

### CD4^+^ T cells expressing GARP/LAP make IL-10 but not IFN-γ

**Figure 3:**
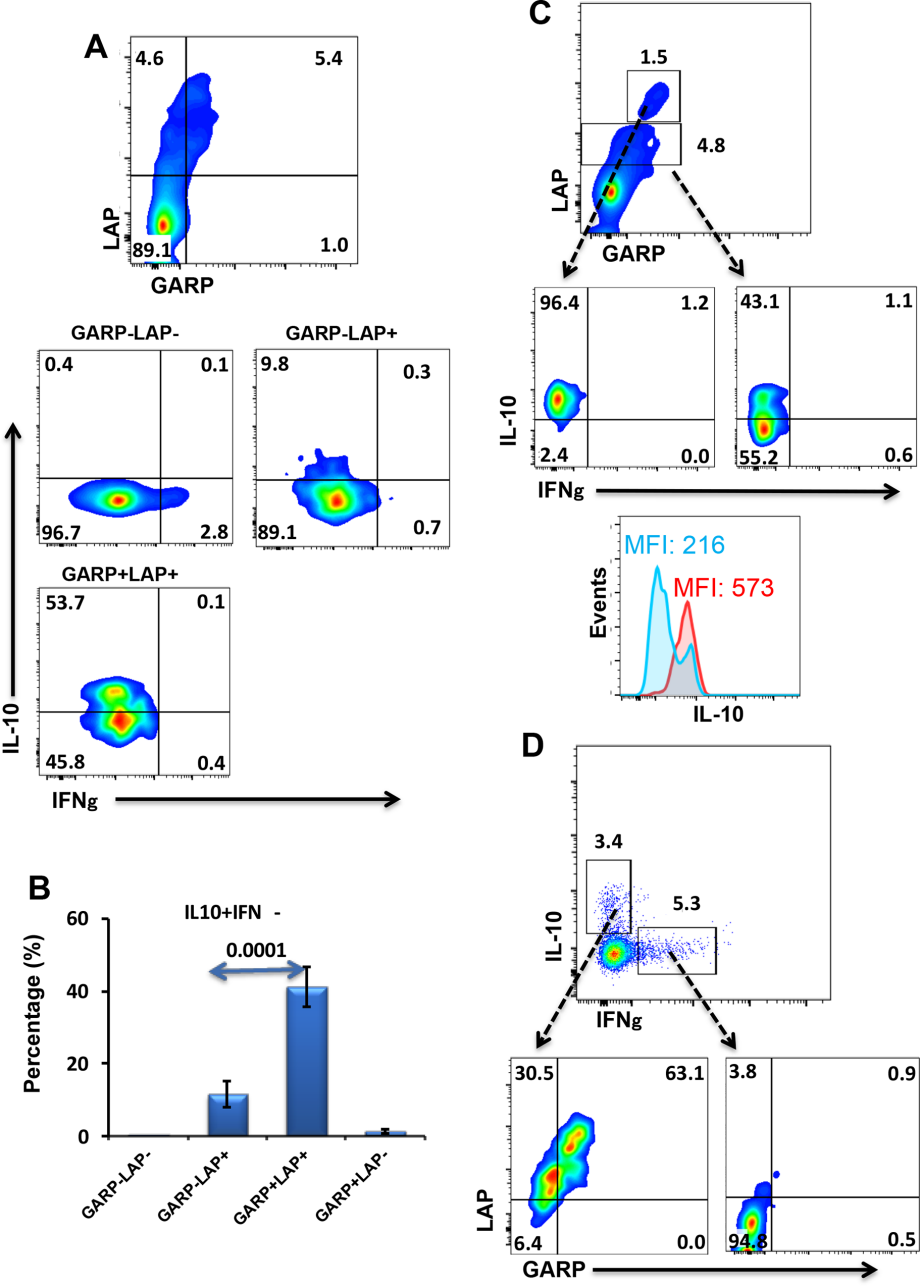
Intracellular cytokine secretion from different GARP^+/−^LAP^+/−^ CD4^+^ T-cell subsets **A.** Representative flow cytometric plots showing GARP^+/−^LAP^+/−^ T-cell subsets and cytokine release (IFNγ and IL-10) from these subsets following PBMCs activation. **B.** Mean percentage ± SEM of IL-10-secreting cells within GARP^+/−^LAP^+/−^ CD4^+^ T-cell subsets in activated PBMCs isolated from 10 healthy donors. **C.** Flow cytometric plots showing that some CD4^+^ T cells express higher levels of GARP/LAP, and all GARP^high^/LAP^high^ cells secreted IL-10, compared to GARP^inter^/LAP^inter^. The overlaid histogram plot shows that MFI of IL-10 expression is higher within GARP^high^/LAP^high^ cells (red) than GARP^inter^/LAP^inter^ cells (blue). **D.** Representative flow cytometric plots show the secretion of IL-10 and IFN-γ from CD4^+^ T cells; IL-10-secreting CD4^+^ T cells express GARP/LAP, while IFN-γ-secreting CD4^+^T cells lack the expression of GARP/LAP.

We then investigated the nature of cytokines released from different subpopulations expressing or lacking GARP/LAP. PBMCs were stimulated by anti-CD3/28 for 18–20 hours to allow induction of GARP/LAP. We noticed GARP/LAP could not be induced on cell surface if Golgi Plug is added during activation period; therefore Golgi Pulg was added for only extra 4 hours to allow detection of cytokines. GARP^−^LAP^−^ CD4^+^ T cells made IFN-γ effector cytokine but not IL-10 immunosuppressive cytokine (Figure 3A). On the other hand, GARP^+^LAP^+^ and GARP^−^LAP^+^ CD4^+^ T cells made IL-10 but not IFN-γ (Figure 3A & 3B). Interestingly, GARP^+^LAP^+^ cells secreted significantly higher levels of IL-10 (41.3 ± 5.4%), compared with GARP^−^LAP^+^ cells (11.5 ± 3.7%), as shown in Figure 3B. We noticed that some CD4^+^ T cells express higher levels of GARP/LAP. Of note, when GARP^high^/LAP^high^ cells were gated, almost all of them secreted IL-10, compared to cells expressed GARP/LAP at intermediate level (Figure 3C). We further confirmed these findings by performing back gating. Figure 3D shows that TCR-stimulated CD4^+^ T cells secrete both IL-10 and IFN-γ. CD4^+^IL-10^+^ T cells expressed GARP/LAP, while CD4^+^IFN-γ^+^ T cells lacked the expression of GARP/LAP. CD8^+^ T cells secreted IFN-γ but not IL-10, and GARP/LAP were not expressed on IFN-γ^+^ or IFN-γ^−^ CD8^+^ T cells (not shown).

### FoxP3^+^Helios^+^ Tregs make IL-10 while FoxP3^+^Helios^−^ Tregs make IL-10 and effector cytokines

**Figure 4:**
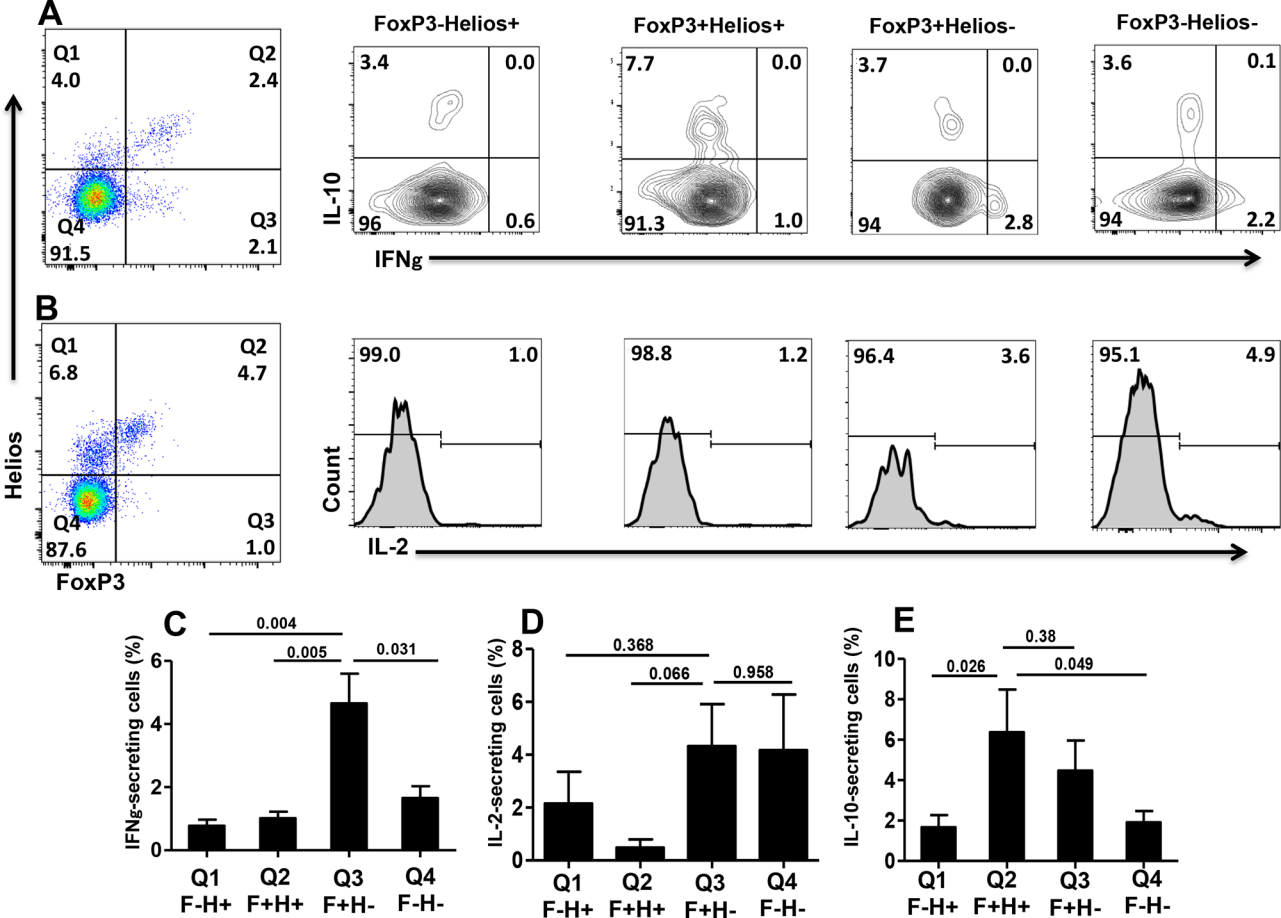
Intracellular cytokine secretion from different FoxP3^+/−^Helios^+/−^ T-cell subsets Representative flow cytometric plots showing FoxP3^+/−^Helios^+/−^ T-cell subsets and intracellular cytokine secretion of IFNγ and IL-10 **(A)** and IL-2 **(B)** from these different subsets following PBMCs activation. Mean percentage ± SEM of IFNγ (**C.** *n* = 9), IL-2- (**D.** *n* = 6) and IL-10-secreting cells (**E.** *n* = 8) within the different FoxP3^+/−^Helios^+/−^ CD4^+^T-cell subsets.

To further elucidate the nature of different FoxP3^+/−^Helios^+/−^ T-cell subsets with respect to cytokine secretion, we investigated the ability of TCR-stimulated PBMCs to release different cytokines including IL-10, as an immunosuppressive cytokine, and IFN-γ and IL-2, as effector cytokines. Proportions of cytokine-secreting cells were not as high as in previous studies because cells were stimulated with anti-CD3/CD28 and not by PMA/ionomycin to allow induction of GARP/LAP. We found that effector cytokines were secreted mainly by FoxP3^+^Helios^−^ Tregs (IFN - γ: 4.7 ± 0.94%; IL - 2: 4.3 ± 1.6%) and FoxP3^−^Helios^−^ conventional CD4^+^ T cells (IFN-γ: 1.7 ± 0.94%; IL-2: 4.3 ± 1.6%) (Figure 4). FoxP3^+^Helios^−^ Tregs contained significantly higher proportions of IFN-γ^+^ cells, compared with all other subpopulations (Figure 4C). In addition, FoxP3^+^Helios^−^ Tregs contained some proportions of IL-10^+^ cells (4.5 ± 1.5%, Figure 4E). Secreting a mixture of effector and immunosuppressive cytokines could be interpreted as FoxP3^+^Helios^−^ Tregs containing a mixture of bona fide Tregs and conventional Teff that were induced to express FoxP3 without being real Tregs. On the other hand, FoxP3^+^Helios^+^ Tregs, the subpopulation expressing GARP/LAP in the activated setting (as described in Figure 2), contained the highest levels of IL-10^+^ cells (6.4 ± 2.1%, Figure 4E), and did not make IFN-γ or IL-2 (Figure 4C & 4D, 4E).

### FoxP3^+^Helios^+^ Tregs have less proliferative ability than FoxP3^+^Helios^−^ Tregs

**Figure 5:**
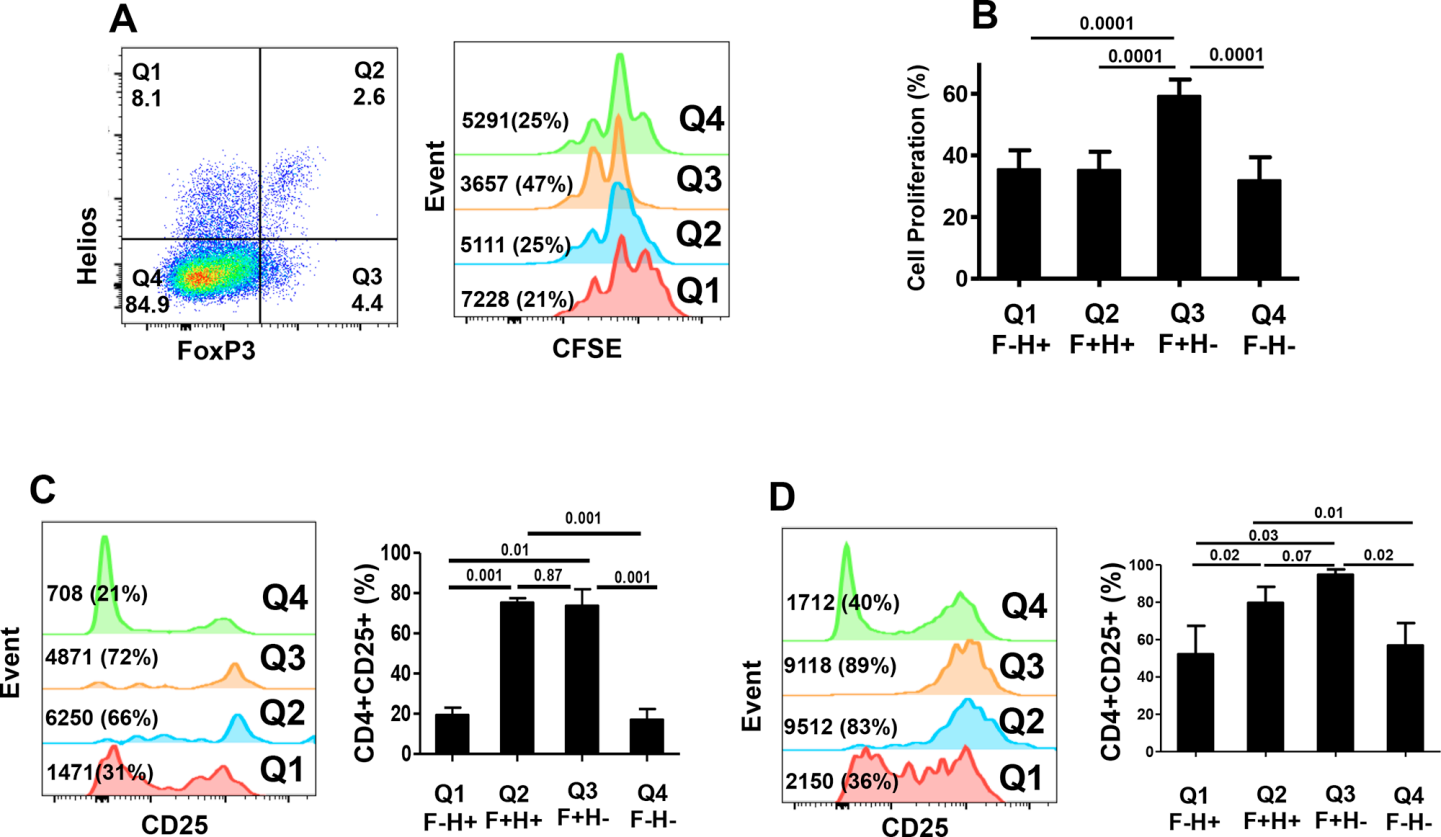
CFSE-based proliferation assays and CD25 expression within different FoxP3^+/−^Helios^+/−^ T-cell subsets **A.** Representative flow cytometric plots showing FoxP3^−^Helios^−^, FoxP3^−^Helios^+^, FoxP3^+^Helios^+^ and FoxP3^+^Helios^−^ T-cell subsets (first plot) and their proliferation (second plot) as measured by CFSE loss. **B.** Mean percentage ± SEM of cell proliferation of these different subsets in PBMCs isolated from 9 healthy donors. Flow cytometric plots and mean percentage ± SEM of CD25 expression in non-activated **(C)** and activated **(D)** FoxP3^+/−^Helios^+/−^ T-cell subsets in PBMCs isolated from 5 healthy donors.

To get further insights into the nature of different FoxP3^+/−^Helios^+/−^ T-cell subsets, we investigated their ability to proliferate in response to TCR stimulation. We set up crude proliferation assays in which all cell populations in PBMCs were kept. This allows the release of IL-2 from TCR-stimulated CD4^+^ T effector cells that can be used by cells expressing the high affinity IL-2R α chain (CD25). We found that FoxP3^+^Helios^−^ CD4^+^ Tregs proliferated significantly higher (59.2 ± 5.4%, Figure 5A & 5B) than all other subpopulations, which showed similar proliferation levels (32–35%, Figure 5B). Having less proliferation capability in response to TCR stimulation even in the presence of IL-2 secreted by effector cells confirms that FoxP3^+^Helios^+^ are more anergic, a characteristic of bona fide Tregs. We measured levels of CD25 expression before and after TCR stimulation to determine the role of IL-2 in inducing proliferation of different subsets. As expected, without activation FoxP3^+^Helios^+^ and FoxP3^+^Helios^−^ Tregs showed constitutive expression of significantly higher levels of CD25 (75.2 ± 2.3% and 73.7 ± 8.2%, respectively; Figure 5C), compared with FoxP3^−^Helios^+^ and FoxP3^−^Helios^−^ subsets (19.4 ± 3.7% and 17.1 ± 5.3%, respectively; Figure 5C). Following TCR stimulation, CD25 was induced on FoxP3^−^Helios^+^ (52.2 ± 15.2%, Figure 5D), FoxP3^+^Helios^−^ (94.8 ± 2.8%, Figure 5D), and FoxP3^−^Helios^−^ (56.8 ± 12.0%, Figure 5D), but not on FoxP3^+^Helios^+^ Tregs, which showed similar levels of CD25 as per before stimulation (79.7 ± 8.5%, Figure 5D). The highest expression of CD25 was detected on FoxP3^+^Helios^−^ Tregs, confirming their higher proliferative capability compared to other subsets. Although, FoxP3^+^Helios^+^ and FoxP3^+^Helios^−^ Tregs expressed similar levels of CD25 prior to stimulation, CD25 was further induced on only FoxP3^+^Helios^−^ Tregs, which proliferated significantly higher.

## DISCUSSION

In this study, we investigated the differences between FoxP3^+/−^Helios^+/−^ CD4^+^ T cell subsets. These included GARP/LAP expression, proliferative capacity and cytokine secretion. GARP and LAP are promising late-stage Treg activation markers that are selectively up-regulated on activated Tregs but not on Teff. GARP and LAP also play functional roles in Treg suppressive activity, making them particularly useful as Treg markers *in vitro* and *in vivo* [13–15, 24]. We here report that, in the steady state, GARP and LAP are expressed on FoxP3^−^Helios^+^ CD4^+^ T cell subset significantly more than CD4^+^FoxP3^+^ Tregs, regardless of Helios expression. We also report, for the first time, that in the activated setting, GARP and LAP are expressed on FoxP3^+^Helios^+^ and FoxP3^−^Helios^+^ T cell subsets. They are expressed at significantly lower levels on FoxP3^+^Helios^−^ CD4^+^ T cells, and not on FoxP3^−^Helios^−^ T cells. These findings indicate that Helios, and not FoxP3, is the key marker to identify activated Tregs, expressing GARP and LAP.

We investigated specific cellular characteristics associated with suppression within different FoxP3^+/−^Helios^+/−^ T-cell subsets following activation. We found that FoxP3^+^Helios^+^ Tregs exhibit more immunosuppressive characteristics, compared with FoxP3^+^Helios^−^ Tregs. These characteristics included expression of GARP/LAP, secretion of immunosuppressive IL-10, lack of effector cytokine secretion (IFN-γ and IL-2) and lower proliferative capacity. FoxP3^+^Helios^+^ Tregs contained the highest levels of IL-10^+^ cells (6.4 ± 2.1%, Figure 4E), and did not secrete significant levels of effector cytokines. In contrast, FoxP3^+^Helios^−^ T cells secreted effector cytokines, and contained the highest proportions of IFN-γ^+^ cells. This FoxP3^+^Helios^−^ T cell subset also contained IL-10^+^ cells (4.5 ± 1.5%, Figure 4E) potentially indicating a mixture of bona fide Tregs, and Teff transiently induced to express FoxP3. The FoxP3^+^Helios^−^ T cell subset has previously been shown to comprise a mixture of T cells secreting IL-10, IL-2, IFN-γ and IL-17 [33]. They have also been reported to comprise a significantly larger proportion of non-suppressive T cell clones compared to FoxP3^+^Helios^+^ Tregs [39]. In line with our findings, other groups have also reported that effector cytokine secretion is mainly limited to the FoxP3^+^Helios^−^ T cell subset, while FoxP3^+^Helios^+^ Tregs are non-cytokine producers, both in healthy and autoimmune disease settings [33, 39–42].

While it is recognized that two distinct subsets of Helios^+^ and Helios^−^ Tregs exist within FoxP3^+^ Tregs, their role and function are still subject to debate and Helios is currently not regarded as a definitive marker to differentiate between pTregs and tTregs [32]. Very recently, in a Helios reporter mice, it was shown that Helios^+^ Tregs have superior ability than Helios^−^ Tregs to suppress T-cell proliferation and cytokine production [29]. Our findings suggest that Helios defines a more suppressive Treg subset. This must however be confirmed in further mechanistic assays. As suggested earlier, FoxP3^+^Helios^−^ Tregs might simply contain a mixture of non-suppressive and suppressive T cells rather than being intrinsically less suppressive than FoxP3^+^Helios^+^ Tregs. Indeed, human Helios^+^ and Helios^−^ Tregs have previously been reported to exhibit a similar suppressive capacity although Helios^−^ secreted greater levels of IFN-γ [32]. The different rates of proliferation reported in our study must also be interpreted carefully; although Tregs show an anergic phenotype *in vitro*, they proliferate at a much higher rate *in vivo* [43].

The FoxP3^−^Helios^+^ T cell population did not secrete significant levels of IL-10, IL-2 or IFN-γ and showed a proliferative capacity comparable to that of FoxP3^+^Helios^+^ Tregs. We can speculate that these might be TGF-β-dependent Th3 cells. Expression of GARP and LAP, as surrogate markers for TGF-β, on these non-cytokine secreting T cells could further support the case for Th3 cells being present. Helios has also been reported to be up-regulated in Th2 and follicular T helper cells (Tfh) independently of FoxP3 [44]. Further studies investigating the cytokine secretion profile of GARP/LAP expressing T cells with FoxP3 and Helios are required.

In accordance with other reports identifying GARP^+^LAP^+^ Tregs as highly immunosuppressive Tregs, we found that GARP^+^LAP^+^ cells secrete IL-10 but not IFN-γ. A distinct GARP^high^LAP^high^ subset with over 90% of cells secreting IL-10 was also identified (Figure 3C). We noticed that following TCR activation, within FoxP3^−^Helios^+^ T cells, there is a subset expressing high level of LAP (Figure 2A & 2B). In contrast, FoxP3^+^Helios^+^ cells mainly increased GARP expression with LAP increased to a lower extent (Figure 2A). This could indicate that FoxP3 inhibits LAP expression or perhaps that, following activation, FoxP3^+^Helios^+^ Tregs release TGF-β from the cell surface. Indeed Tregs utilize TGF-β as a suppressive mechanism. One recent murine study reported that GARP expression was induced on FoxP3^+^ T cells following 24 hours of *in vitro* activation, while LAP expression was only induced after 48–72 h of activation [45]. A FoxP3^−^LAP^+^ Treg subset was also identified in humans [23]. These LAP^+^ Tregs secreted IL-10, IFN-γ and TGF-β upon activation but were not studied for expression of GARP and Helios [23].

When interpreting these results, we should take into account the significant functional and phenotypic heterogeneity and plasticity that Tregs exhibit [12, 46, 47]. Numerous tissue- and antigen-specific Treg subsets with distinct features exist, including follicular Tregs, ‘ex-Tregs’, and IL-17-secreting Tregs. Immune regulation involves complex interplay and cross-talk between Treg subsets, Teff, DCs and other immune cells. Identification of highly suppressive individual Treg subsets is important. However, these subsets should be considered in the overall context of physiological or pathological immune functioning. Impact of Treg subsets on clinical outcomes can vary considerably [5, 48]. For example, immunotherapies enhanced Helios expression in CD4^+^ Tregs in the responder group of rheumatoid arthritis patients [49]. In contrast, in SLE patients, levels of Helios^+^ Tregs were significantly increased and correlated positively with SLE disease activity [41, 42].

Therapeutic modalities for treating autoimmune diseases, allergies and graft rejection should be designed to induce and/or expand the most suppressive Treg subsets. Our work suggests these might be the FoxP3^+^Helios^+^GARP^+^LAP^+^ Tregs. Recent work on a method for generating human allo-antigen specific Tregs was able to induce large-scale generation of suppressive Tregs all of which expressed FoxP3, Helios, GARP and LAP [50]. On the other hand, when treating cancers and infectious diseases, therapeutic modalities should avoid the generation and/or expansion of FoxP3^+^Helios^+^ Tregs to avoid compromising anti-tumor or anti-microbial immune responses.

Further investigations into levels and functions of FoxP3^+/−^Helios^+/−^ T cell subsets as well as correlations with clinical outcomes in different disease settings should provide important insights for understanding the nature and role of these subsets. Utilizing GARP and LAP as cell surface markers for identifying highly suppressive FoxP3^+^Helios^+^ Tregs may enable effective isolation of highly suppressive Treg subsets, in the activated setting, both for therapeutic manipulation and further downstream investigations into Treg biology and diversity.

## MATERIALS AND METHODS

### Cell isolation and preparation

Whole blood samples were either collected from healthy donors or were obtained from UK National Blood Service. Ethical approvals were obtained prior to samples' collection. Peripheral blood mononuclear cells (PBMCs) were isolated from whole blood using Ficoll-Hypaque (Sigma-Aldrich, UK) density gradient centrifugation. PBMCs were then frozen at 5–10 × 10^6^ cells/ml in cryovials in 1 ml of freezing media (50% FCS, 40% RPMI-1640 and 10% DMSO) and stored in liquid nitrogen (LN) for later use. Trypan blue was used for PBMC viability testing and counting.

### GARP and LAP expression on Treg subpopulations

PBMCs were thawed and suspended at 2 × 10^6^ cells/well in 2 ml complete medium [RPMI-1640 supplemented with L-glutamine 2 mM, 10% FCS, Streptomycin 100 μg/ml and Penicillin 100 Units/ml] in a 24-well non-treated culture plate. PBMCs were either plated as non-activated in non-coated wells or activated in pre-coated wells with plate-bound 2 μg/ml anti-CD3 antibody (clone OKT3 clone, eBioscience, Hatfield, UK) and 2 μg/ml anti-CD28 antibody (CD28.2 clone, eBioscience). Plated cells were incubated for 18–20 hours in a humidified incubator at 37°C 5% CO2. Cells were collected and blocked for FcR with IgG from human serum (Sigma-Aldrich, UK) and were then stained for extracellular markers using mouse anti-human CD4-PerCP-Cy5.5 (eBioscience), mouse anti-human CD3-APC-H7 (BD Biosciences, Oxford, UK), mouse anti-human GARP-APC (BD Biosciences), and mouse anti-human LAP-PE (BD Biosciences). For intracellular markers, cells were subsequently fixed, permeabilized and blocked using rat serum (eBiosceince) and mouse serum (Sigma-Aldrich) before staining with rat anti-human FoxP3-PE-Cy7 (PCH101 clone, eBioscience) and Armenian hamster anti-mouse/human Helios-FITC (22F6 clone, Biolegend, Cambridge, UK). Following two further permeabilization washes, cells were re-suspended in flow cytometry buffer. Fixation, permeabilization and flow cytometry buffers were all from eBioscience or BD Biosciences and prepared as per the manufacturer's instructions. Flow cytometric data was acquired on a FACSVerse or FACSCanto II flow cytometers (BD Biosciences, USA). Data analysis was performed using BD FACSuite or FlowJo version x 10.0.7r2 software.

### Cytokine release from different Treg subpopulations

Thawed PBMCs were plated in complete medium in a 24-well non-treated culture plate pre-coated with 2 μg/ml anti-CD3 and 2 μg/ml anti-CD28. To investigate IFN-γ and IL-10 release from subpopulations expressing GARP/LAP or not, cells were incubated for 24 hours at 37°C 5% CO2 and 1 μg/ml Golgi Plug (BD Biosciences) was added for the last 4 hours of activation. Cells were first stained for extracellular markers using mouse anti-human CD4-PerCP-Cy5.5, mouse anti-human CD3-APC-H7, mouse anti-human GARP-APC, and mouse anti-human LAP-PE. For intracellular cytokines, cells were subsequently fixed, permeabilized and blocked using mouse serum before staining with anti-human IL-10-FITC (eBioscience) and mouse anti-human IFNγ-PE-Cy7 (BD Pharmingen, BD Biosciences, UK).

In another experimental panel to investigate IFN-γ and IL-10 release from different subpopulations expressing FoxP3/Helios or not, cells were activated for 24 hours, with Golgi Plug added for the last 4 hours, then stained for extracellular markers using mouse anti-human CD4-PerCP-Cy5.5 and mouse anti-human CD3-APC-H7. For intracellular transcription factors/cytokines, cells were subsequently fixed, permeabilized and blocked using rat and mouse serum before staining with rat anti-human FoxP3-PE (eBioscience) and Armenian hamster anti-mouse/human Helios-FITC, together with rat anti-human IL-10-APC (BD Pharmingen) and mouse anti-human IFNγ-PE-Cy7. To investigate IL-2 release, cells were activated for 6 hours, with Golgi Plug added for the last 4 hours, followed by staining with rat anti-human IL-2-PE-Cy7 (eBioscience) and flow cytometric and data analysis were performed as described above.

### CFSE-based proliferation assays

Proliferation of different subsets was measured by carboxyfluorescein diacetate succinimidyl ester (CFSE)-based proliferation assays. Briefly, PBMCs were suspended at 1 × 10^6^ cells/ml in pre-warmed 0.1% BSA in PBS and incubated with 0.25 μM CFSE/ml (eBioscience) for 10 minutes at 37°C, followed by a series of washes. CFSE-labeled cells were suspended in complete medium and were polyclonally stimulated with plate-bound 2 μg/ml anti-CD3 and 2 μg/ml anti-CD28. Cells were incubated for 3 days in a humidified incubator at 37°C 5% CO_2_. CFSE-labeled cells were then stained for surface markers using anti-CD3-APC-H7 and anti-CD4-PerCp-5.5 antibodies, followed by intracellular staining using anti-FoxP3-PE-Cy7 and anti-Helios-APC antibodies. In another experimental setting, CD25 expression in different FoxP3/Helios CD4^+^ T-cell subsets was determined using mouse anti-human CD25-APC-H7 (BD Biosciences). Proliferation and various marker expressions in different subsets were evaluated by flow cytometry.

### Statistical analysis

Statistical analysis was performed using GraphPad Prism 5.0 software (GraphPad Software, United States). Paired *T* test was used to test for differences within groups. *P* value ≤ 0.05 was considered statistically significant. The data are presented as means ± SEM.
